# Molecular Screening of 43 Brazilian Families Diagnosed with Leber Congenital Amaurosis or Early-Onset Severe Retinal Dystrophy

**DOI:** 10.3390/genes8120355

**Published:** 2017-11-29

**Authors:** Fernanda B. O. Porto, Evan M. Jones, Justin Branch, Zachry T. Soens, Igor Mendes Maia, Isadora F. G. Sena, Shirley A. M. Sampaio, Renata T. Simões, Rui Chen

**Affiliations:** 1 INRET Clínica e Centro de Pesquisa, Belo Horizonte, 30150290 Minas Gerais, Brazil; fernandabop@gmail.com (F.B.O.P.); shirley.msampaio@yahoo.com.br (S.A.M.S.); 2 Centro Oftalmológico de Minas Gerais, COMG, Belo Horizonte, 30150290 Minas Gerais, Brazil; 3 Department of Molecular and Human Genetics, Baylor College of Medicine, Houston, TX 77030, USA; evanj@bcm.edu (E.M.J.); zachrysoens@gmail.com (Z.T.S.); 4 Human Genome Sequencing Center, Baylor College of Medicine, Houston, TX 77030, USA; justin.branch@bcm.edu; 5 Instituto de Ensino e Pesquisa da Santa Casa de Belo Horizonte, IEP/SCBH, Belo Horizonte, 30150290 Minas Gerais, Brazil; igormde@gmail.com (I.M.M.); isadorafgs@hotmail.com (I.F.G.S.); simoesrt@yahoo.com.br (R.T.S.); 6 Structural and Computational Biology & Molecular Biophysics, Baylor College of Medicine, Houston, TX 77030, USA

**Keywords:** retina, genetics, ophthalmology, Leber congenital amaurosis, early-onset retinal dystrophy, next-generation sequencing

## Abstract

Leber congenital amaurosis (LCA) is a severe disease that leads to complete blindness in children, typically before the first year of life. Due to the clinical and genetic heterogeneity among LCA and other retinal diseases, providing patients with a molecular diagnosis is essential to assigning an accurate clinical diagnosis. Using our gene panel that targets 300 genes that are known to cause retinal disease, including 24 genes reported to cause LCA, we sequenced 43 unrelated probands with Brazilian ancestry. We identified 42 unique variants and were able to assign a molecular diagnosis to 30/43 (70%) Brazilian patients. Among these, 30 patients were initially diagnosed with LCA or a form of early-onset retinal dystrophy, 17 patients harbored mutations in LCA-associated genes, while 13 patients had mutations in genes that were reported to cause other diseases involving the retina.

## 1. Introduction

Inherited retinal dystrophies are a large heterogeneous group of diseases that are characterized by a degeneration of the light sensitive photoreceptor cells that are found in the retina. Some disorders lead to damage in both the rod and cone photoreceptor cells, leading to a complete loss of vision while others cause the specific loss of central or peripheral vision. Among this group of dystrophies is Leber congenital amaurosis (LCA), a primarily autosomal recessive disease, which is considered to be the most common cause of childhood blindness that affects both rod and cone photoreceptors [1]. LCA is estimated to affect 1 in 50,000 individuals and accounts for 5% of all retinal dystrophies and 20% of blindness in school-age children. The disease typically manifests within the first year of life and is characterized by minimal if any vision beyond infancy, congenital nystagmus, normal fundus appearance initially or various fundus changes, and a minimal or non-detectable signal on an electroretinogram (ERG) [2]. Despite these defined features, the clinical phenotype and genetic cause of LCA largely overlaps with that of other retinal dystrophies, which creates challenges in providing patients with a definitive clinical and molecular diagnosis [1,3]. For example, the clinical features of early-onset severe retinal dystrophies (EOSRD), such as juvenile retinitis pigmentosa (RP), can be similar to those of LCA. In 1869 Theodor Leber, the first to describe a child with LCA, initially diagnosed the disease as a congenital form of RP [4]. An abnormal recording on an ERG was later used to be an essential classifier of LCA [5]. In a later publication in 1916, Leber described a milder form of the same disease that manifests in the 6th and 7th years of life and led to blindness by 30 years of age, which he considered to be on the same spectrum as LCA. The disease has since been referred to as EOSRD [6]. Some patients fulfill the criteria for LCA but retain relatively good vision and better fit the definition of EOSRD. Therefore, the classification of these patients is not always clear. Moreover, juvenile RP and cone-rod dystrophy (CORD) tend to possess a milder phenotype than LCA in that vision loss is progressive [7]. The genetic overlap among retinal dystrophies can also complicate the diagnostic process in that variants in a particular set of genes may be associated with multiple retinal phenotypes [1]. Variants in several LCA-associated genes (*CRX*, *CRB1*, *IMPDH1*, *RDH12*, *RPE65*, *TULP1*, *SPATA7*) have been implicated to cause juvenile RP and CORD [2,3]. Furthermore, retinal dystrophies can be syndromic, in which non-retinal features are reported. Such syndromic dystrophies that cause LCA or an LCA like phenotype include Alström syndrome [8,9], Bardet-Biedl syndrome [10], and Senior-Loken syndrome [9,11]. Due to the genetic heterogeneity of LCA, a molecular diagnosis is essential for providing a patient with an accurate prognosis of disease, genetic counseling, and access to genetic therapies. While there exists no effective cure for retinal dystrophies, currently growing gene therapy clinical trials will potentially offer a remedy for inherited retinal disease patients [12,13].

To date, there are 24 genes that have been associated with LCA, in which next-generation sequencing (NGS) of the protein coding regions has led to a molecular diagnostic rate of about 75% [2]. In addition, previous studies have shown that the mutation spectrum differs among different populations [14,15,16]. The goal of this study was to perform a molecular screening of 43 unrelated probands of Brazilian ancestry initially diagnosed with LCA or EOSRD, which so far has been understudied. To evaluate the variant spectrum, a NGS capture panel that targets and sequences the exons of all known retinal disease genes has been developed. Using this targeted approach, we have previously sequenced multiple patient cohorts with retinal disease, including a Chinese cohort of 145 families that were diagnosed with LCA, leading to a solving rate of 76.6% [2]. In the study reported herein, our capture panel approach was able to achieve a solving rate of 70%, in which we identified variants in a total of 30 Brazilian probands. Among these 30 probands, 17 were solved by variants found in genes that have been reported to cause LCA while the remaining 13 probands were solved by variants in genes that were reported to cause other retinal diseases. Based upon our molecular diagnosis of these 13 probands, we were able to refine the clinical diagnosis of four patients, which reveals the importance of combining genetic and clinical information to provide retinal disease patients with an accurate diagnosis. Here, we report the results of the first comprehensive NGS genetic analysis of a Brazilian cohort diagnosed with either LCA or EOSRD.

## 2. Materials and Methods 

### 2.1. Patient Recruitment and Diagnostic Criteria

All Brazilian probands underwent an ophthalmological examination at the INRET Clínica e Centro de Pesquisa and/or Centro Oftalmológico de Minas Gerais, Brazil. Participants in this study originated from various locations in Brazil. We defined LCA/EOSRD as severe visual impairment within the first year after birth, nystagmus, oculodigital sign, and severely reduced or non-detectable ERG. When possible, a clinical assessment of family members was performed, and a sample of DNA was obtained. Informed consent was acquired from all of the patients and participating family members. All of the procedures were approved by appropriate institutional review boards, ethics committees (Santa Casa de Belo Horizonte Research Ethics Committee CAAE: 11168613.0.0000.5138), and adhered to the tenets of the declaration of Helsinki. All of the participants underwent detailed ophthalmologic examinations, including best-corrected visual acuity (BCVA), slit-lamp biomicroscopy, dilated-pupil fundus examination, cycloplegic refraction, full-field ERG, flash visual evoked potential, optical coherence tomography (OCT), fundus photographs, and autofluorescence, depending on the patient age. DNA was extracted from patient blood using miniDNA QIAmp Kit (QIAGEN, Hilden, Germany), according to the manufacturer’s protocol. The quantity and quality of the DNA was verified using NanoDrop (ThermoFisher Scientific, Waltham, MA, USA).

### 2.2. Capture Panel Design and Library Preparation

We sequenced 300 genes that were associated with retinal disease for each patient using a capture panel that our group previously developed (Supplementary Table S1). The panel also includes the frequent pathogenic intronic variant c.2992+1655A>G that has been established to create a donor splice site within intron 26 of *CEP290*, causing the inclusion of a cryptic exon and a premature stop codon [17].

For capture sequencing, we generated paired-end libraries, according to the manufacturer’s protocol. We began by shearing 1 ug of genomic DNA into 300–500 base pair (bp) fragments and by adding an adenosine base to the 3′ end using the Klenow exonuclease. Y-shaped index adaptors were ligated to the fragments and 8–10 cycles of PCR amplification were performed for each sample. The DNA libraries were quantified using the PicoGreen fluorescence assay (ThermoFisher Scientific) and 50 ng of the generated libraries were used for each capture reaction. NimbleGen SeqCap EZ (Roche, Pleasanton, CA, USA) Hybridization and Wash kits were used for washing and recovering captured DNA. The captured DNA was quantified and was sent for sequencing using an Illumina HiSeq 2000 machine, following the manufacturer’s protocol. Illumina sequencing was performed at Baylor College of Medicine’s Human Genome Sequencing Center (Houston, TX, USA).

### 2.3. Bioinformatic Analysis

To process the data from capture sequencing, we used a variant calling, filtering, and annotation pipeline designed by our group to discover retinal disease-causing variants. Using the Burrows-Wheeler Aligner (BWA) algorithm, sequencing reads were aligned to the human genome reference version hg19. Base quality scores were recalibrated and reads realigned using the Genome Analysis Toolkit (GATK). Single nucleotide variants (SNVs) and small insertions and deletions (InDels) were called using GATK. Because LCA is a rare disease, we removed variants with a minor allele frequency greater than 0.5%, as these occur too frequently to cause Mendelian retinal disease. Variant frequencies were obtained from a merged database including the Exome Aggregation Consortium (ExAC), the UK10K project, the Kyoto University’s Human Genetic Variation Database (HGVD), and our internal exome database of 55,000 individuals. 

We consider multiple criteria when evaluating the potential pathogenicity of a variant and classify our variants into three categories with different clinical significance according to the American College of Medical Genetics and Genomics: (1) variants found in the Human Gene Mutation Database. (HGMD) database that have been well established to cause disease; (2) variants that present with a clear loss-of-function (LoF) consequence, such as nonsense, frameshift, and splicing variants that are located at the invariable two base pairs flanking an exon, and (3) nonsynonymous variants that have not been previously reported in retinal disease patients but are predicted to be deleterious by in silico prediction algorithms [18]. All of the variants must match their corresponding gene’s inheritance pattern, be validated using Sanger sequencing, and pass segregation tests using family members when available. The database of nonsynonymous functional predictions (dbNSFP) was used to compile pathogenicity predictions, and a majority consensus of the algorithms was used to determine deleteriousness. 

### 2.4. Sanger Validation 

For each identified pathogenic variant, primers were designed using Primer3 [19] to amplify the exon containing the variant and at least 50 flanking base pairs, or to produce an amplicon at least 200 base pairs in size centered around the variant. PCR amplicons were sequenced on an ABI 3730XL or 3500XL Genetic Analyzer. Sequencing results were analyzed using Sequencher5.0 software (Gene Codes Corporation, Ann Arbor, MI, USA).

## 3. Results

### 3.1. Patient Geographic Distribution and Sequencing

DNA was collected from a total of 43 unrelated families whose probands matched our diagnostic criteria. Families were recruited from around Belo Horizonte and the state of Minas Gerais with the exception of five probands (FBP_9, FBP_34, FBP_60, FBP_62, FBP_174) who traveled roughly 3000 km to the city of Belo Horizonte for evaluation. 

Utilizing our capture panel technology, we were able to obtain an average of 2.75 million paired reads per sample, in which 49% were mapped to the target region, resulting in a 71× mean per base coverage for the targeted regions. Moreover, an average of 92% of targeted regions had 10× coverage or more, which was sufficient for accurate variant calling. The pipeline initially called an average of 3356 SNVs and 494 InDels for each sample. After filtering against control databases, we were able to narrow our search to an average of 10 rare SNVs and 2 InDels for each patient sample.

### 3.2. Identifying Pathogenic Variants 

**Figure 1:**
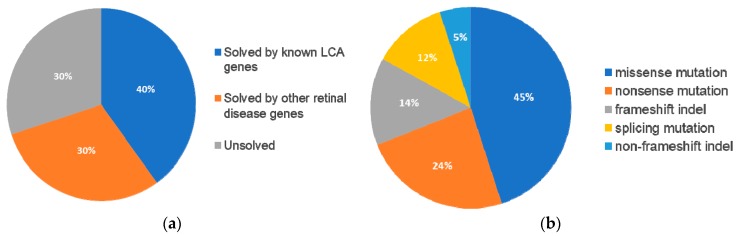
Breakdown across 43 probands with pathogenic variants in known retinal disease genes and the respective type classification for these mutations. (**A**) 17 (40%) individuals were solved by pathogenic variants in known Leber Congenital Amaurosis (LCA)-associated genes while 13 (30%) of individuals were solved by pathogenic variants in genes not typically associated with LCA. 13 (30%) of individuals were not identified to contain likely pathogenic mutations in known retinal disease genes. (**B**) The percentage of each classification type of mutation across the 42 pathogenic variants identified in the 30 solved cases.

Following our pathogenic variant identification guidelines (see methods), putative pathogenic variants were identified in 30/43 (70%) patients that carried variants in LCA-associated genes and other retinal disease genes (Figure 1, Supplementary Table S2). We identified 42 unique variants, of which, 27 (64%) were reported and 15 (36%) were novel. Of these 42 variants, the majority were missense variants, followed by nonsense and other type of mutations (Table 1).

### 3.3. Patients with Variants in Established Leber Congenital Amaurosis-Associated Genes 

We identified 17 (40%) patients that were diagnosed with LCA or EOSRD that harbor variants in genes that have been reported in RetNet [20] to cause LCA/EOSRD (Table 2).

Among the 17 patients with variants in known LCA-associated genes, *CRB1*, *RPE65*, and *CEP290* were the most frequently mutated genes, pathogenic variants being identified in three patients each (Table 2). Two variants were found to be recurrent within this patient group. The first variant, identified in three unrelated probands (FBP_29, FBP_62, and FBP_173), is the *CEP290* intron 26 cryptic exon-forming variant (c.2991+1655A>G). The second recurrent variant is a *CRB1* (p.Cys948Tyr) variant, which is found in two of our Brazilian probands (FBP_36 and FBP_174).

**Figure 2:**
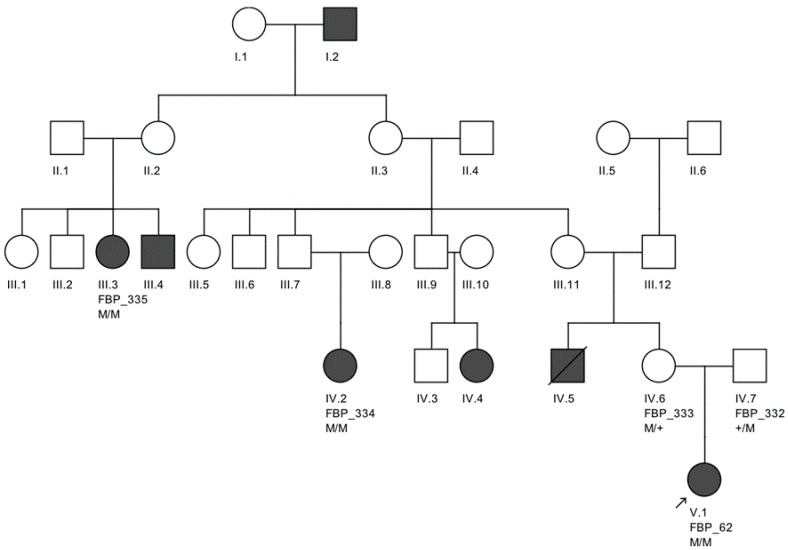
Large Brazilian family of proband FBP_62 from Belo Horizonte, sequenced for the frequent founder intronic variant M (c.2991+1665A>C) in *CEP290*. Affected individuals FBP_334, FBP_335, and FBP_62 were all confirmed homozygous for this variant. Non-affected family members FBP_333 and FBP_332 were confirmed heterozygous carriers for this variant.

The largest pedigree for which we were able to obtain familial information was for proband FBP_62, who was homozygous for the *CEP290* intronic variant. We went on to Sanger validate the genotype of this variant as homozygous in two additional affected family members, implicating its contribution to disease in a total of six family members affected by LCA (Figure 2). 

**Figure 3:**
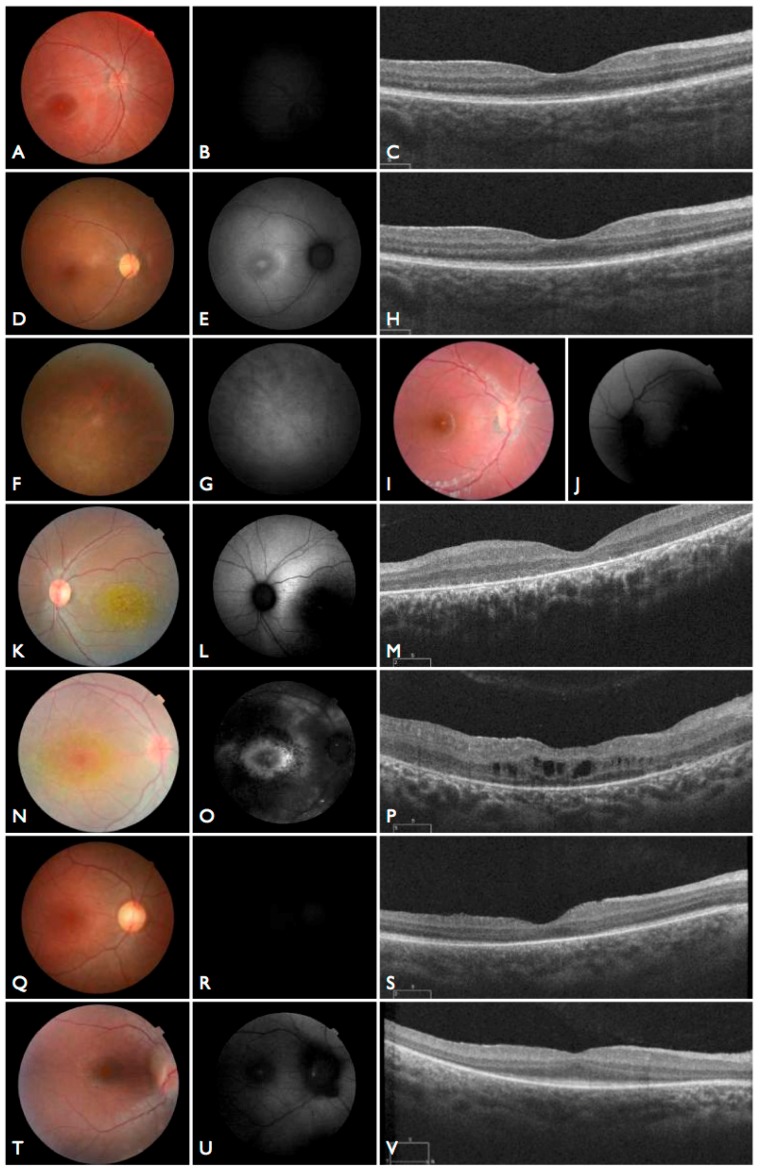
Fundus and optical coherence tomography (OCT) images for seven individuals solved by mutations in canonical LCA-associated genes. (**A**–**C**) Fundus and OCT images of FBP_ABC solved by mutations in *RPE65*. (**D**–**H**) Fundus and OCT images of FBP_29 solved by mutations in *CEP290*. (**I**,**J**) Fundus images of FBP_4 solved mutations in *GUCY2D*. (**K**–**M**) Fundus and OCT images of FBP_34 solved by compound heterozygous mutations in *CRB1*. (**N**–**P**) Fundus and OCT images of FBP_36 solved by mutations in *CRB1*. (**Q**–**S**) Fundus and OCT images of FBP_55 solved by mutations in *LRAT*. (**T**–**V**) Fundus and OCT images of FBP_62 solved by a homozygous mutation in *CEP2*.

Moreover, we confirmed that the intronic mutation is heterozygous in both parents. Clinical data from FBP_62 is consistent with a *CEP290* causal genotype (Figure 3T–V), including the severe but stable vision loss. At the age of six, visual acuity was hand motion both for right eye (RE) and left eye (LE). Full-field ERG was non-recordable. OCT showed remnant macular photoreceptors despite severe functional impairment. Additionally, there was noted photoreceptor loss surrounding the cone-rich fovea. Autofluorescence revealed right eye optic drusen and augmented signal at the foveas.

We note an interesting phenotype in FBP_29, who harbored reported compound heterozygous splicing variants in *CEP290*: a splicing variant that was reported to cause Joubert syndrome and the recurrent *CEP290* deep intronic cryptic splice site variant. She was adopted at five months of age with strabismus and nystagmus. She was diagnosed with retinal dystrophy at three years old. Her biological parents were non-consanguineous. She had one paternal uncle who was affected, and nine siblings, of which two brothers were affected. One of the affected brothers presented with mental retardation, aside from ocular defects. The proband FBP_29 is remarkable for the preservation of central vision. When evaluated at age 19, the visual acuity (VA) was RE 20/30 (+3.00sph ‒3cyl 15°) and LE 20/30 (+1.75sph ‒3.00cyl 165°). Goldman visual fields were constricted with 10° tunnel vision (V-4-e). Full-field ERG disclosed non-recordable responses of rods and cones. Fundus examination showed bilateral optic disc pallor, severe attenuated retinal vessels, and no pigmentation (Figure 3D–H). Autofluorescence revealed an augmented autofluorescent ring contouring the fovea. Interestingly, the autofluorescence disclosed para-arterial retinal pigment epithelium (RPE) preservation. The OCT showed retention of the photoreceptor and inner laminar architecture in the central retinal with surrounding photoreceptor loss. 

**Figure 4:**
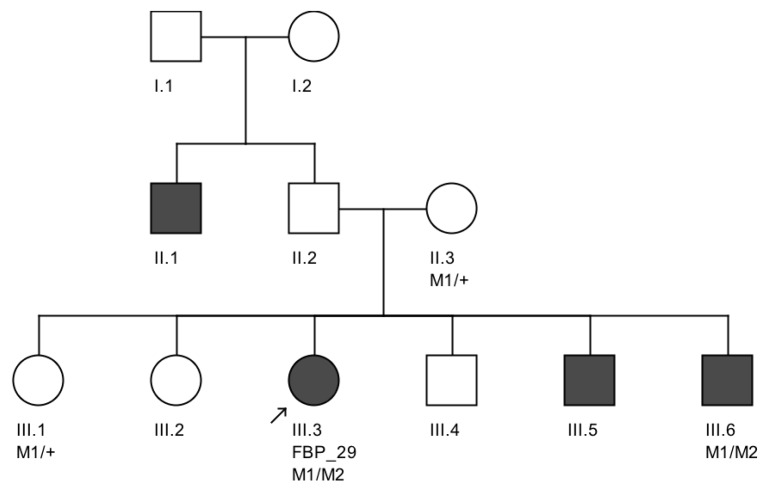
Variant segregation in the family of proband FBP_29. These *CEP290* splicing variants M1 (c.6271-8 T>G) and M2 (c.C2991+1655A>G) segregated in trans in which M1 was maternally inherited. Both of the affected siblings that were available for segregation testing (III.3 and III.6) were verified to be heterozygous for each variant.

We further conducted co-segregation tests and identified the splicing mutation c.6271-8T>G is maternally inherited, and the affected brother of the proband also carries the same compound heterozygous mutations (Figure 4). Interestingly, he presented also with mental retardation, which was not present in FBP_29 or the other affected brother. Unfortunately, we were not able to obtain cranial Magnetic Resonance Imaging (MRI).

**Figure 5:**
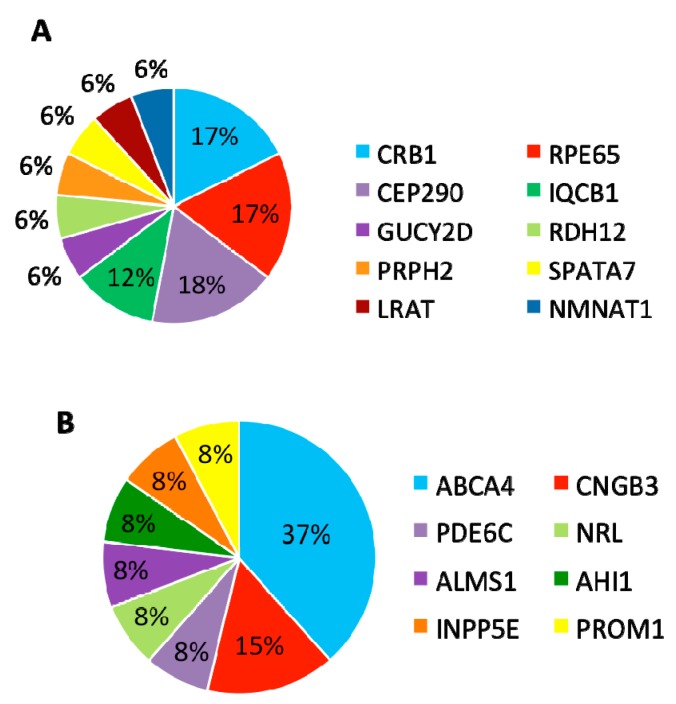
Distribution of genes mutated in 30 Brazilian probands solved by either canonical LCA/EOSRD genes or genes associated with other retinal diseases. (**A**) Percentage of genes with causal variants in the 17 probands solved by variants in canonical LCA/EOSRD genes. (**B**) Percentage of genes with causal variants in the 13 probands solved by variants in genes associated with other retinal diseases.

In summary, mutations were identified in 8 genes that have been associated with LCA and *CRB1*, *RPE65*, and *CEP290* are the most frequently mutated genes (Figure 5A).

### 3.4. Patients with Variants in Other Retinal Disease Genes

Furthermore, 13 (30%) patients carried variants in genes that have been associated with other retinal diseases than LCA (Table 3). Among these 13 patients, eight carried compound heterozygous variants, four carried homozygous variants, and one patient carried compound homozygous variants. A total of 19 variants were identified in eight different retinal disease genes that consisted of 12 reported and seven novel variants. Of this group of patients, *ABCA4* was the most frequently mutated gene, seen in five patients (Figure 5B). The most frequent variant in this group was the missense variant c.C1804T (p.R602W) in *ABCA4* present in three patients. The missense variant c.T1622C (p.L541P) in *ABCA4* and small deletion c.1148delC (p.T383fs) in *CNGB3* were carried by two patients.

### 3.5. Reassessment of Patients with Variants in Other Retinal Disease Genes

We observed 13 (30%) patients carrying causal variants in disease genes that have not been linked to non-syndromic LCA previously. We reassessed clinical data of these patients and revisited the clinical phenotypes for 8 of them. 

### 3.6. ABCA4

In our study, we found five patients with seven previously reported variants in ABCA4. The earliest diagnoses were made in the patients FBP_1 and FBP_61. The other three patients (FBP_9, FBP_65, FBP_131), despite describing important visual loss since the first years of life, did not have an ERG performed at younger age (respectively, at 10, 14, and 19 years old). These patients were classified as EOSRD.

**Figure 6:**
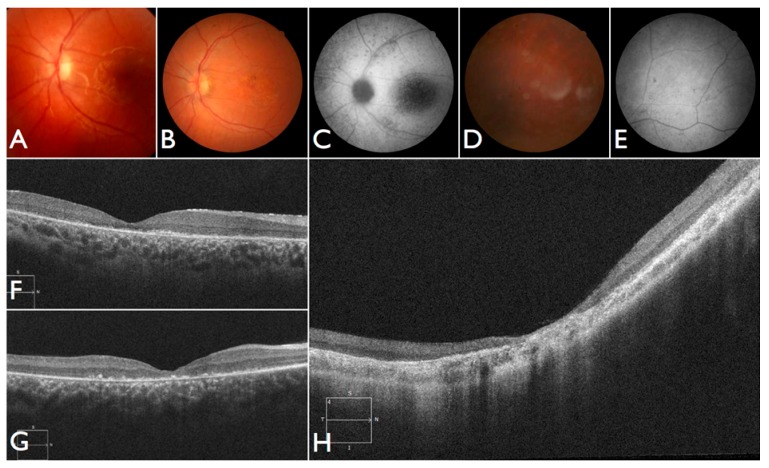
Fundus and OCT images for four individuals solved by pathogenic variants in *ABCA4*. (**A**–**E**) Fundus and autofluorescence images of FBP_1; (**F**) OCT image ofFBP_61. (**G**) OCT image of FBP_65. (**H**) OCT image of FBP_9.

The patient FBP_1 presented strabismus, loss of vision and night blindness at four years old, when she began wearing visual correction. The first specific exams were performed at seven years old when visual acuity was 20/200 both eyes and only subtle macular changes were observed (Figure 6A). The scotopic and photopic ERG were non-recordable ERG. She complained that the vision was consistently worse in scotopic conditions. Because of the early and severe phenotype, the scarcity of findings in the fundus she was classified as EOSRD. At nine years old she presented fine granularity of the macular pigment epithelium (Figure 6B). Fundus autofluorescence was abnormally high, with some punctate loss of fundus autofluorescence along the arcades and areas hypo- and hyper-autofluorescence being limited to the macula area and from the arcades to the periphery (Figure 6C–E), smaller and with more granular appearance than the flecks and maculopathy observed in Stargardt disease.

Patient FBP_61 was referred because of night blindness, visual field difficulties, and the school reported he has had difficulty seeing the blackboard over the last year. He is a five-year-old boy, with optic disc pallor, vessel attenuation, and RPE atrophy. ERG was non-recordable. The OCT scan showed thinned retina with relatively preserved outer nuclear leayer in the central fovea, but no EZ band or ROS were detectable. Outside the fovea there is outer retinal structure loss (outer nuclear layer (ONL), ellipsoid zone band (EZB), rod outer segement (ROS)) (Figure 6F). Based on the phenotype, characterized by severe and early difuse retinal degeneration, with no recordable ERG, RPE atrophy, vessel attenuation, and optic disc pallor at five years of age, this patient was classified as early-onset retinal dystrophy.

### 3.7. CNGB3

In our Brazilian cohort, we identified two individuals with variants in *CNGB3*. Patient FBP_171 was homozygous for the p.Thr383fs* variant. She was referred because of nystagmus and poor visual contact at three months. The retina showed dull foveal reflex. At six months, un-sedated photopic and scotopic ISCEV ERG were non-recordable. 

**Figure 7:**
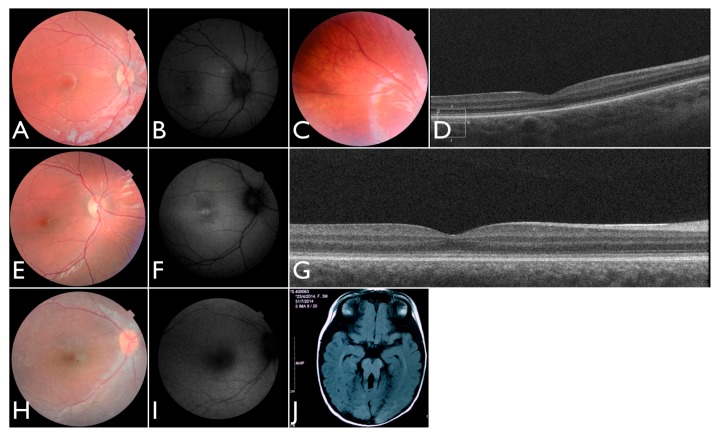
Images for four individuals solved by mutations in other retinal disease genes. (**A**–**C**) Fundus, autofluorescence and OCT images of FBP_three solved by mutations in *CNGB3*. (**D**–**G**) Fundus and autofluorescence images of FBP_171 solved by mutations in *CNGB3*. (**H**–**I**) Fundus image and autofluorescence of FBP_170 solved by mutations in *ALMS1*. (**J**) Axial T1 MRI of FBP_327 solved by mutations in the AHI1 showing the molar tooth sign.

After identifying the homozygous *CNGB3* variant, the patient was again examined and at the age of 18 months the un-sedated ERG testing showed again no discernable photoreceptor response. She is now three years old, wearing filtered glasses and presenting normal neurologic development. Retinal images show a subtle macular change and translucent RPE (Figure 7A–D). An oval area of reduced autofluorescence was observed in the fovea. The OCT scan discloses flattening and disruption in the ellipsoidal zone at the fovea, thinner outer segments and spared interdigitation zone.

Patient FBP_3 was heterozygous for the p.Thr383fs* and p.Arg203* variants in *CNGB3*. He presented nystagmus and severe photophobia since birth, keeping his eyes closed most of the time. He was first examined at four years old. Visual acuity was 5/50 (+2.00 sph) for both RE and LE. A full-field ERG was performed without sedation and disclosed no discernable scotopic and photopic responses. Retinas were fairly normal, and fundus autofluorescence imaging disclosed reduced foveal autofluorescence surrounded by a zone of increased autofluorescence. At eight years old, the ERG was confirmed to be non-recordable (Figure 7E–G). The OCT shows intact outer retinal structure except for a flattening and a subtle discontinuity of the ellipsoid zone.

In summary, we documented early rod involvement in two patients with the *CNGB3* mutations. 

### 3.8. Reassessment of Patients with PDE6C, ALMS1, AHI1, and INPP5E Variants

In our study we found one patient with a novel homozygous variant in *PDE6C*. She presented progressive visual loss and severe photophobia since early in life. Nystagmus was first observed at the age of three. ERG was recorded at five years old and showed significantly reduced but measurable rod responses, and non-recordable cone ERG. The clinical phenotype was consistent with severe early childhood onset cone-rod dystrophy. The novel p.Val810* homozygous *PDE6C* variant may be related to the severe phenotype. 

Additionally, we identified one individual with biallelic LoF variants in *ALMS1* diagnosed with EOSRD, obesity, and hypotonia [21]. She was born from a non-consanguineous family with two other normal children. She presented nystagmus, vision loss and photophobia since birth. At the age of four, ophthalmic exam revealed high hyperopia (RE +9.00sph −2.00cyl × 175°; LE + 10.00sph −2.00cyl 175°) optic disc hyperemia; subtle retinal vessel attenuation (Figure 7H,I), and non-recordable ERG. *COH1* gene sequencing was negative. At that age, there were no renal, cardiac, or endocrinal abnormalities. 

One of our patients had compound heterozygous variants in *AHI1*, one of which has been previously reported and occurs within the WD40 domain. She was the first daughter of a non-consanguineous marriage. At five months of age, the ERG was non-recordable and a blood sample was collected for genetic testing. She had visual loss, nystagmus, and developmental delay. We re-examined the patient once after analysis of the gentic testing results. An MRI elucidated molar tooth sign (Figure 7J) and an abdominal ultrasound revealed right renal agenesia.

**Figure 8:**
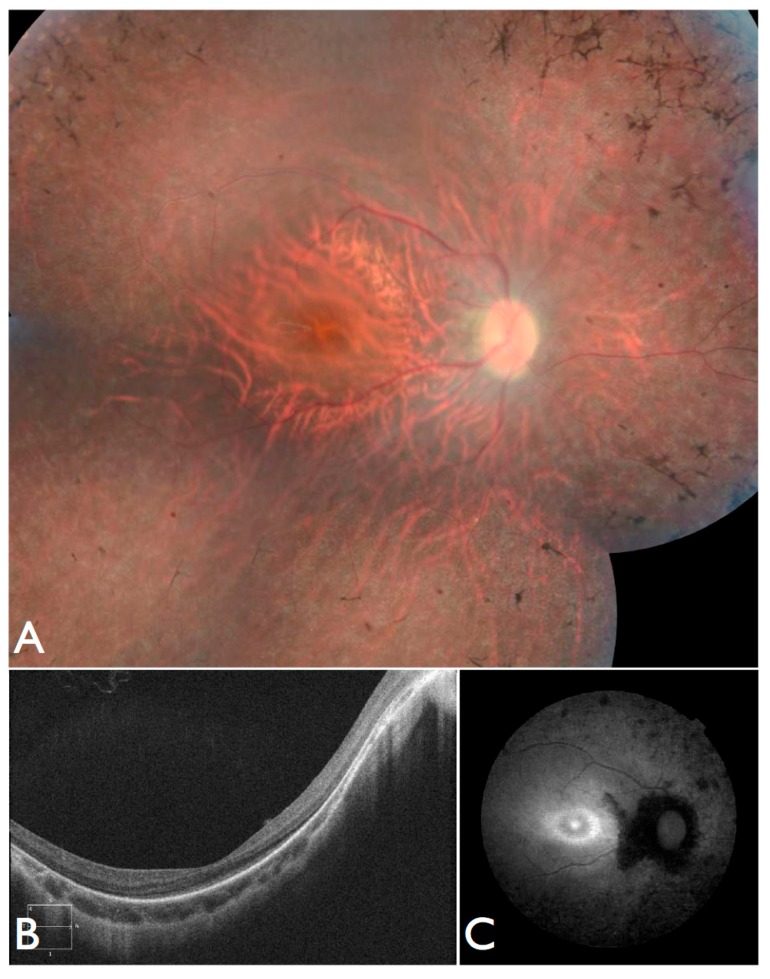
Fundus and OCT images for FBP_53, solved by mutations in *INPP5E*. (**A**) Color fundus montages showing bilateral pale optic discs, attenuated retinal vessels, normal foveal reflexes, and mild desaturation of the fundus coloration with macular preservation and pigmentary disturbance at the periphery. (**B**) OCT scan and (**C**) Retinal autofluorescence.

Lastly, we identified an individual compound heterozygous for variants in *INPP5E*. The patient’s first symptoms were strabismus, visual field defects, and low vision, wearing glasses since the age of three. At the age of 21, he complains of night blindness. Nystagmus, neurologic, or extraocular manifestations were never present. BCVA was 5/50 RE (−9.25sph −2.25cyl 5°) and 5/80 LE (−9.00sph −2.75cyl 175°). ERG was non-recordable Fundus images exhibited retinal vessel attenuation, optic disc pallor, RPE atrophy with bone-spicule pigment deposits and relative macular sparing (Figure 8A), and a hyper-autofluorescent ring (Figure 8B). OCT revealed photoreceptor loss in the periphery and relatively preserved retina in the central area. Choroidal and retinal thickness were diminished, and the posterior wall was bowed posteriorly with the most protruded point away from the central fovea (Figure 8C). After analyzing the genetic testing results, we re-examined the patient and noted no systemic manifestations. 

### 3.9. Comparison of Brazilian Cohort to Chinese and European Individuals Diagnosed with LCA

To determine how similar our Brazilian cohort is to other LCA populations, we compared our results to our previously sequenced Chinese cohort of 131 families with known LCA-associated gene mutations (14 individuals in the original 145 indiviual cohort carried pathogenic mutations in other retinal disease genes) [2] and to various European studies of LCA individuals that were solved by known LCA-associated gene mutations [22]. In our comparison, we included the 17 patients solved by variants in LCA-associated genes and 11 unsolved patients that possessed clinical features of LCA. Therefore, we considered a total of 28 patients in our comparison that had the classical clinical features of LCA. Among these 28 patients, three had variants in *CRB1* (11%), three in *RPE65* (11%), three in *CEP290* (11%), and one patient with a variant in *GUCY2D* (4%). 

In comparison to the Chinese LCA population, in which the four most frequently mutated genes were *CRB1* (16.8%), *GUCY2D* (10.7%), *RPGRIP1* (7.6%), and *CEP290* (6.9%). In regards to our study, we noted that our Brazilian cohort carried more variants in *RPE65* (11% vs. 4.6%) and *CEP290* (11% vs. 6.9%). 

In comparison to the European population in which the four most frequently mutated genes were *CEP290* (15%), *GUCY2D* (11.7%), *CRB1* (9.9%), and *IMPDH1* (8.3%) In our study, we found our Brazilian cohort to carry more variants in *CRB1* (11% vs. 9.9%) and *RPE65* (11% vs. 6%). The European population harbored more variants in *GUCY2D* (11.7% vs. 4%). While the Europeans carried more variants in *CEP290* (15%) than both Brazilian (11%) and Chinese (6.9%) cohorts. 

Interestingly, the two previously reported founder variants found in *CRB1* (p.Cys948Tyr) and *CEP290* (c.2991+1655A>G) identified in our Brazilian cohort were present in the European LCA population but absent in the Chinese population.

## 4. Discussion

We report herein the mutation screening of 43 LCA families, the first Brazilian LCA cohort so far to our knowledge. We used a capture panel-based NGS method and a panel that targets 300 known retinal disease genes, including 24 genes that are reported to cause LCA. The study of these 43 families, together with other recent studies, provide insight into the genetic basis of Brazilian LCA patients, which previously were unstudied. 

*CRB1*, *RPE65*, and *CEP290* were the most frequently mutated LCA-associated genes in this Brazilian cohort with two variants being recurrent, both of which have previously been reported to be founder mutations with worldwide prevalence [22]. The first of these being the *CEP290* intron 26 cryptic exon-forming variant (c.2991+1655A>G), which is present in 20% of patients of European descent. The second recurrent variant is the *CRB1* (p.Cys948Tyr). The presence of both of these variants within our Brazilian cohort lends support to previous founder effect observations, and the introduction of each to the Brazilian population could be due to the influx of European ancestral alleles during the colonial era. The FBP_62 family is from Maranhão, a Brazilian state that is 2.5 km from Minas Gerais that was invaded and settled by the French in the seventeenth century. The other patients with variants in *CRB1*, *RPE65*, and *CEP290* came from the state of Minas Gerais, which was colonized by the Europeans, primarily Portuguese, during the colonial era. 

Despite LCA as a well-characterized disease, the severity of clinical manifestations may vary. EOSRD is a milder form of the same disease. Furthermore, the clinical phenotype and genetic cause of LCA largely overlaps with that of other retinal dystrophies, creating challenges in providing patients with a definitive clinical and molecular diagnosis. In the cohort we describe herein, patient FBP_29 had milder than typical LCA phenotype. We identified two known heterozygous mutations in the *CEP290* gene for this patient, who fulfills some of the criteria for LCA, having poor vision and nystagmus in the first year of life. However, her disorder is remarkable for the development and retention of relatively good central vision well into the second decade of life. Thus, her condition better fits the definition of EOSRD. It is possible that the combination of these two heterozygous mutations in this subject, both of which affect splicing but outside of the canonical splicing site, still allow for the translation of protein with enough residual function, as opposed to a complete homozygous null allele, resulting in a milder, EOSRD, phenotype. 

On the other hand, in our cohort, some patients presented an earlier and severe visual loss. We present herein five patients with the diagnosis of EOSRD related to *ABCA4* with non-recordable ERG, night-blindness, and important visual impairment early in the first years of life. Mutations in *ABCA4* may lead to many different phenotypes, vastly heterogeneous clinical manifestations, and disease course. Stargardt disease, RP, CORD, age-related macular degeneration, fundus flavimaculatus, and EOSRD have been reported to be caused by variants in *ABCA4* [23,24,25,26,27,28]. The patients we present were classified as EOSRD, a known phenotype among the various phenotypes already known in conjunction with *ABCA4*. Because of the early and severe visual impairment, they were within the LCA/EOSRD cohort. Recently, another LCA cohort identified a patient with compound heterozygous *ABCA4* variants and the pre-test diagnosis of LCA, presenting early night vision loss and non-recordable ERG [2]. Eventually different combinations of *ABCA4* alleles and different genetic background are related to severe and heterogeneous phenotypes as the patients we report on.

Similarly, patient FBP_35 exhibited an early and severe phenotype with no measurable photopic ERG and almost non-recordable rod ERG at the age of five. We identified a homozygous novel variant in the *PDE6C* gene. *PDE6C* mutations are described to cause cone dystrophy. Rod involvement is not a major consequence of *PDE6C* mutations, although some dysfunction of rods is reported to occur later in life [29]. When comparing the phenotype and genotype of this patient, we found that we were facing an EOSRD. In addition, the diagnosis of the rare clumped pigmentary retinal degeneration was established in a patient that was previously diagnosed with LCA. Therefore, our results suggest that the initial clinical diagnosis may not be perfect, and that molecular diagnosis can be a useful tool to refine or offer an early and precise clinical diagnosis. 

This becomes even more important when the initial presentation was predominantly ocular and with genetic testing we were able to establish the diagnosis of systemic diseases and anticipate important systemic manifestations. In our study, one patient was diagnosed with Joubert syndrome 3 (MIM:608629) at six months of age. She was the first daughter of the non-consanguineous marriage, and the ocular manifestation allowed molecular diagnosis of a systemic disease and appropriate genetic counseling. Furthermore, the initial symptoms of a systemic disease can be inexplicit, making diagnosis difficult. The molecular diagnosis allowed for us to diagnose Alström syndrome in a context when only indistinct symptoms were present. Due to the visual impairments seen within the first year of life, patients with Alström syndrome can be mistaken as having non-syndromic retinal disease such as LCA, and only later developing hearing loss, obesity, and diabetes mellitus.

In our study, we identified two families with variants in *CNGB3*. *CNGB3* mutations have been associated with achromatopsia and juvenile macular degeneration [30]. We reviewed all of the exams and clinical history, and also invited the patients to be reevaluated. The patients with the diagnosis of LCA that we examined showed significant visual impairment, especially in photopic conditions, they benefited from filtered glasses, and the autofluorescence exam disclosed central maculopathy, similarly to the achromatopsia phenotype. The non-recordable rod and cone ERGs were repeated and confirmed. The initial LCA diagnosis was appropriate and novel genotype-phenotype correlations due to the different nature of alleles and/or different genetic background is possible. Importantly, as the full-field ERG was the distinguishing feature between LCA and achromatopsia that is related to *CNGB3*, the early assessment of patients with full-field ERG will potentially identify the frequency of the LCA phenotype in *CNGB3* related retinal dystrophy.

Interestingly, we also identified one individual harboring two heterozygous *INPP5E* variants. The inositol polyphosphate 5-phosphatase (*INPP5E*) mutations cause Joubert syndrome 1 (MIM:213300), a clinically and genetically heterogeneous group of disorders that are characterized by midbrain-hindbrain malformation and various associated ciliopathies that include retinal dystrophy, nephronophthisis, liver fibrosis, and polydactyly. The patient we report herein was revisited and no extraocular manifestation was found, suggesting that some patients may never develop the systemic complications that are usually associated with *INPP5E* mutations. 

In summary, we successfully identified pathogenic variants in 30 Brazilian families among 43 total LCA families (70%). The clinical heterogeneity that exists among retinal phenotypes can lead to a difficulty in providing a precise clinical diagnosis due to an overlap of ophthalmic findings. Novel genotype-phenotype correlations that have not been previously reported may exist within the Brazilian population. For the 13 (30%) patients unsolved by retinal disease gene capture sequencing, we plan to perform whole exome sequencing to potentially discover novel gene associations with retinal disease. 

## Figures and Tables

**Table 1:** Composition of pathogenic variants identified in the 30 Brazilian probands.

	Known	Novel	Total
Missense	14	5	19
Nonsense	8	2	10
Frameshift indel	2	4	6
Splicing	2	3	5
Non-frameshift indel	1	1	2
	17 (64%)	15 (36%)	42

**Table 2:** 17 unrelated Brazilian probands diagnosed with LCA or early-onset retinal dystrophy and solved by pathogenic mutations in canonical LCA/ early-onset severe retinal dystrophies (EOSRD) disease genes.

Family	Patient ID	Gene	HGMD RefSeq ID	Genotype	gDNA Change	cDNA Change	Protein Change	HGMD Pubmed ID
1	FBP_ABC	*RPE65*	NM_000329.2	Homozygous	chr1:68910339 G>A	c.370C>T	p.Arg124*	20683928; 24066033
2	FBP_EFG	*RPE65*	NM_000329.2	Heterozygous	chr1:68903976 A>G	c.1022T>C	p.Leu341Ser	15837919
			NM_000329.2	Heterozygous	chr1:68910540 C>T	c.272G>A	p.Arg91Gln	11095629; 19431183
4	FBP_2	*RPE65*	NM_000329.2	Homozygous	chr1:68906619 C>T	c.560G>A	p.Gly187Glu	Novel
6	FBP_4	*GUCY2D*	NM_000180.3	Homozygous	chr17:7909997 C>A	c.1343C>A	p.Ser448*	10951519
7	FBP_8	*RDH12*	NM_152443.2	Heterozygous	chr14:68191305 C>T	c.184C>T	p.Arg62*	15258582
			NM_152443.2	Heterozygous	chr14:68195947 T>A	c.698T>A	p.Val233Asp	20683928
9	FBP_29	*CEP290*	NM_025114.3	Heterozygous	chr12:88494960 T>C	c.2991+1655A>G	p.Cys998*	16909394; 19823873; 23591405
			NM_025114.3	Heterozygous	chr12:88456563 A>C	c.6271−8T>G	p.?	17409309
10	FBP_30	*IQCB1*	NM_001023570.2	Homozygous	chr3:121527857 C>T	c.394−1G>A	p.?	Novel
12	FBP_34	*CRB1*	NM_201253.2	Heterozygous	chr1:197446848 G>A	c.4060G>A	p.Ala1354Thr	11389483; 15459956
			NM_201253.2	Heterozygous	chr1:197390718 G>A	c.1760G>A	p.Cys587Tyr	15459956
		*PROM1*	NM_006017.2	Homozygous	chr4:16024948 C>A	c.784+1G>T	p.?	Novel
14	FBP_36	*CRB1*	NM_201253.2	Heterozygous	chr1:197403836 G>A	c.2843G>A	p.Cys948Tyr	10508521; 20956273; 23379534
			NM_201253.2	Heterozygous	chr1:197390591 T>C	c.1633T>C	p.Ser545Pro	Novel
15	FBP_48	*PRPH2*	NM_000322.4	Homozygous	chr6:42689937 G>A	c.136C>T	p.Arg46*	8111389; 20213611; 23105016
16	FBP_49	*SPATA7*	NM_018418.4	Heterozygous	chr14:88892899 C>CT	c.697dupT	p.Leu232fs	Novel
			NM_018418.4	Heterozygous	chr14:88892905 TAACA>T	c.703_706del	p.235_236del	Novel
19	FBP_55	*LRAT*	NM_004744.3	Homozygous	chr4:155665824 T>C	c.346T>C	p.Phe116Leu	Novel
20	FBP_57	*NMNAT1*	NM_022787.3	Heterozygous	chr1:10035827 T>G	c.293T>G	p.Val98Gly	22842231; 22842227; 22842230
			NM_022787.3	Heterozygous	chr1:10032168 G>A	c.37G>A	p.Ala13Thr	22842229; 22842227; 22842230
24	FBP_62	*CEP290*	NM_025114.3	Homozygous	chr12:88494960 T>C	c.2991+1655A>G	p.Cys998*	16909394; 19823873; 23591405
28	FBP_67	*IQCB1*	NM_001023570.2	Homozygous	chr3:121491467 G>A	c.1504C>T	p.Arg502*	20881296; 23446637
35	FBP_173	*CEP290*	NM_025114.3	Heterozygous	chr12:88494960 T>C	c.2991+1655A>G	p.Cys998*	16909394; 19823873; 23591405
			NM_025114.3	Heterozygous	chr12:88514886 A>C	c.1247T>G	p.Leu416*	Novel
36	FBP_174	*CRB1*	NM_201253.2	Homozygous	chr1:197403836 G>A	c.2843G>A	p.Cys948Tyr	10508521; 20956273; 23379534

HGMD: Human Gene Mutation Database; gDNA: genomic DNA; cDNA: complementary DNA.

**Table 3:** Thirteen unrelated Brazilian Probands diagnosed with LCA or EOSRD and solved by mutations in retinal disease genes not typically associated with LCA.

Family	Patient ID	Gene	HGMD Refseq ID	Genotype	gDNA Change	cDNA Change	Protein Change	HGMD Pubmed ID
3	FBP_1	*ABCA4*	NM_000350.2	Homozygous	chr1:94486888 G>C	c.4926C>G	p.Ser1642Arg	11846518; 23953153; 24713488
			NM_000350.2	Homozygous	chr1:94485275 TCACGCAGATGGCAAC>T	c.5044_5058del	p.1682_1686del	9054934; 23755871; 24713488
5	FBP_3	*CNGB3*	NM_019098.4	Heterozygous	chr8:87680283 G>A	c.607C>T	p.Arg203*	15258582
			NM_019098.4	Heterozygous	chr8:87656008 AG>A	c.1148delC	p.Thr383fs	20683928
8	FBP_9	*ABCA4*	NM_000350.2	Heterozygous	chr1:94471056 G>A	c.6088C>T	p.Arg2030*	9973280; 23755871
			NM_000350.2	Heterozygous	chr1:94508433 G>GAC	c.3211_3212insGT	p.Ser1071fs	9054934; 23755871; 24265693
13	FBP_35	*PDE6C*	NM_006204.3	Homozygous	chr10:95422844 AG>A	c.2428delG	p.Val810*	Novel
18	FBP_53	*INPP5E*	NM_019892.4	Heterozygous	chr9:139327633 C>T	c.1133G>A	p.Arg378His	Novel
			NM_019892.4	Heterozygous	chr9:139324200 C>T	c.G1862G>A	p.Arg621Gln	2303453
22	FBP_60	*PROM1*	NM_006017.2	Homozygous	chr4:15989283 TA>T	c.2130+2del	p.?	Novel
23	FBP_61	*ABCA4*	NM_000350.2	Heterozygous	chr1:94496008 C>T	c.4328G>A	p.Arg1443His	10958763
			NM_000350.2	Heterozygous	chr1:94528266 G>A	c.1804C>T	p.Arg602Trp	9973280; 16103129; 23695285
			NM_000350.2	Heterozygous	chr1:94528806 A>G	c.1622T>C	p.Leu541Pro	9781034; 11017087; 16103129
26	FBP_65	*ABCA4*	NM_000350.2	Heterozygous	chr1:94528266 G>A	c.1804C>T	p.Arg602Trp	9973280; 16103129; 23695285
			NM_000350.2	Heterozygous	chr1:94528806 A>G	c.1622T>C	p.Leu541Pro	9781034; 11017087; 16103129
30	FBP_131	*ABCA4*	NM_000350.2	Homozygous	chr1:94528266 G>A	c.1804C>T	p.Arg602Trp	9973280; 16103129; 23695285
31	FBP_169	*NRL*	NM_006177.3	Heterozygous	chr14:24550743 G>C	c.416C>G	p.Ser139Trp	Novel
			NM_006177.3	Heterozygous	chr14:24550504 AG>A	c.654delC	p.Arg218fs	15591106; 17335001; 22334370
32	FBP_170	*ALMS1*	NM_015120.4	Heterozygous	chr2:73675871 TC>T	c.2215delC	p.Leu739fs	Novel
			NM_015120.4	Heterozygous	chr2:73676715 T>TA	c.3059dupA	p.Tyr1020_Ala1021delins*	Novel
33	FBP_171	*CNGB3*	NM_019098.4	Homozygous	chr8:87656008 AG>A	c.1148delC	p.Thr383fs	10888875; 12815043; 16379026
39	FBP_327	*AHI1*	NM_017651.4	Heterozygous	chr6:135754219 G>A	c.2212C>T	p.Arg738*	16453322; 21866095
			NM_017651.4	Heterozygous	chr6:135777010 AG>A	c.1205delC	p.Pro402fs	Novel

## Supplementary Materials

The following are available online at www.mdpi.com/2073-4425/8/12/355/s1. Table S1: Complete list of (300) gene panel for initial capture sequencing of inherited retinal disease patients and reason for inclusion in panel; Table S2: Comprehensive table of pathogenic variants identified in the 43 unrelated Brazilian probands.

## References

[1] Nash B.M., Wright D.C., Grigg J.R., Bennetts B., Jamieson R.V. (2015). Retinal dystrophies, genomic applications in diagnosis and prospects for therapy. Transl. Pediatr..

[2] Wang H., Wang X., Zou X., Xu S., Li H., Soens Z.T., Wang K., Li Y., Dong F., Chen R. (2015). Comprehensive molecular diagnosis of a large chinese leber congenital amaurosis cohort. Investig. Ophthalmol. Vis. Sci..

[3] Wang F., Wang H., Tuan H.-F.F., Nguyen D.H., Sun V., Keser V., Bowne S.J., Sullivan L.S., Luo H., Zhao L. (2014). Next generation sequencing-based molecular diagnosis of retinitis pigmentosa: Identification of a novel genotype-phenotype correlation and clinical refinements. Hum. Genet..

[4] Leber T. (1869). Ueber Retinitis pigmentosa und angeborene Amaurose. Archiv für Opthalmologie.

[5] Chacon-Camacho O.F., Zenteno J.C. (2015). Review and update on the molecular basis of leber congenital amaurosis. World J. Clin. Cases.

[6] Gregory-Evans K., Pennesi M.E., Weleber R.G., Ryan S.J., Sadda S.R., Hinton D.R., Schachat A.P., Sadda S.R., Wilkinson C.P., Wiedemann P., Schachat A.P., Saunders W.B. (2013). Chapter 40—Retinitis Pigmentosa and Allied Disorders, in Retina.

[7] Wang X., Wang H., Sun V., Tuan H.-F.F., Keser V., Wang K., Ren H., Lopez I., Zaneveld J.E., Siddiqui S. (2013). Comprehensive molecular diagnosis of 179 leber congenital amaurosis and juvenile retinitis pigmentosa patients by targeted next generation sequencing. J. Med. Genet..

[8] Xu Y., Guan L., Xiao X., Zhang J., Li S., Jiang H., Jia X., Yin Y., Guo X., Wang J. (2015). *ALMS1* null mutations: A common cause of leber congenital amaurosis and early-onset severe cone-rod dystrophy. Clin. Genet..

[9] Wang X., Wang H., Cao M., Li Z., Chen X., Patenia C., Gore A., Abboud E.B., Al-Rajhi A.A., Lewis R.A. (2011). Whole-exome sequencing identifies *ALMS1*, *IQCB1*, *CNGA3*, and *MYO7A* mutations in patients with leber congenital amaurosis. Hum. Mutat..

[10] Wang H., Chen X., Dudinsky L., Patenia C., Chen Y., Li Y., Wei Y., Abboud E.B., Al-Rajhi A.A., Lewis R.A. (2011). Exome capture sequencing identifies a novel mutation in BBS4. Mol. Vis..

[11] Estrada-Cuzcano A., Koenekoop R.K., Coppieters F., Kohl S., Lopez I., Collin R.W., De Baere E.B., Roeleveld D., Marek J., Bernd A. (2011). *IQCB1* mutations in patients with leber congenital amaurosis. Investig. Ophthalmol. Vis. Sci..

[12] Jacobson S.G., Cideciyan A.V., Ratnakaram R., Heon E., Schwartz S.B., Roman A.J., Peden M.C., Aleman T.S., Boye S.L., Sumaroka A. (2012). Gene therapy for leber congenital amaurosis caused by *RPE65* mutations: Safety and efficacy in 15 children and adults followed up to 3 years. Arch. Ophthalmol. (Chic. Ill 1960).

[13] Han Z., Conley S.M., Naash M.I. (2014). Gene therapy for stargardt disease associated with *ABCA4* gene. Adv. Exp. Med. Biol..

[14] Wang S., Zhang Q., Zhang X., Wang Z., Zhao P. (2016). Clinical and genetic characteristics of leber congenital amaurosis with novel mutations in known genes based on a chinese eastern coast han population. Graefe’s Arch. Clin. Exp. Ophthalmol..

[15] Musada G.R., Syed H., Jalali S., Chakrabarti S., Kaur I. (2016). Mutation spectrum of the *FZD-4*, *TSPAN12* and *ZNF408* genes in indian fevr patients. BMC Ophthalmol..

[16] Chen X., Sheng X., Sun X., Zhang Y., Jiang C., Li H., Ding S., Liu Y., Liu W., Li Z. (2016). Next-generation sequencing extends the phenotypic spectrum for *LCA5* mutations: Novel *LCA5* mutations in cone dystrophy. Sci. Rep..

[17] Den Hollander A.I., Koenekoop R.K., Yzer S., Lopez I., Arends M.L., Voesenek K.E., Zonneveld M.N., Strom T.M., Meitinger T., Brunner H.G. (2006). Mutations in the *CEP290* (*NPHP6*) gene are a frequent cause of leber congenital amaurosis. Am. J. Hum. Genet..

[18] Richards S., Aziz N., Bale S., Bick D., Das S., Gastier-Foster J., Grody W.W., Hegde M., Lyon E., Spector E. (2015). Standards and guidelines for the interpretation of sequence variants: A joint consensus recommendation of the american college of medical genetics and genomics and the association for molecular pathology. Genet. Med. Off. J. Am. Coll. Med. Genet..

[19] Rozen S., Skaletsky H.J. Primer3. http://biotools.umassmed.edu/bioapps/primer3_www.cgi.

[20] Diager S.P., Sullivan S.L., Bowne S.J., Rossiter B.J.F. Retnet—Retinal Information Network. https://sph.uth.edu/retnet/.

[21] Kolehmainen J., Black G.C., Saarinen A., Chandler K., Clayton-Smith J., Träskelin A.-L.L., Perveen R., Kivitie-Kallio S., Norio R., Warburg M. (2003). Cohen syndrome is caused by mutations in a novel gene, coh1, encoding a transmembrane protein with a presumed role in vesicle-mediated sorting and intracellular protein transport. Am. J. Hum. Genet..

[22] Den Hollander A.I., Roepman R., Koenekoop R.K., Cremers F.P. (2008). Leber congenital amaurosis: Genes, proteins and disease mechanisms. Prog. Retinal Eye Res..

[23] Allikmets R., Singh N., Sun H., Shroyer N.F., Hutchinson A., Chidambaram A., Gerrard B., Baird L., Stauffer D., Peiffer A. (1997). A photoreceptor cell-specific ATP-binding transporter gene (*ABCR*) is mutated in recessive Stargardt macular dystrophy. Nat. Genet..

[24] Allikmets R., Shroyer N.F., Singh N., Seddon J.M., Lewis R.A., Bernstein P.S., Peiffer A., Zabriskie N.A., Li Y., Hutchinson A. (1997). Mutation of the Stargardt Disease Gene (*ABCR*) in Age-Related Macular Degeneration. Science.

[25] Martínez-Mir A., Paloma E., Allikmets R., Ayuso C., del Rio T., Dean M., Vilageliu L., Gonzàlez-Duarte R., Balcells S. (1998). Retinitis pigmentosa caused by a homozygous mutation in the stargardt disease gene abcr. Nat. Genet..

[26] Michaelides M., Chen L.L., Brantley M.A., Andorf J.L., Isaak E.M., Jenkins S.A., Holder G.E., Bird A.C., Stone E.M., Webster A.R. (2007). *ABCA4* mutations and discordant *ABCA4* alleles in patients and siblings with bull’s-eye maculopathy. Br. J. Ophthalmol..

[27] Souied E.H., Ducroq D., Rozet J.M., Gerber S., Perrault I., Sterkers M., Benhamou N., Munnich A., Coscas G., Soubrane G. (1999). A novel *ABCR* nonsense mutation responsible for late-onset fundus flavimaculatus. Investig. Ophthalmol. Vis. Sci..

[28] Maugeri A., Klevering B.J., Rohrschneider K., Blankenagel A., Brunner H.G., Deutman A.F., Hoyng C.B., Cremers F.P. (2000). Mutations in the *ABCA4 (ABCR)* gene are the major cause of autosomal recessive cone-rod dystrophy. Am. J. Hum. Genet..

[29] Thiadens A.A., den Hollander A.I., Roosing S., Nabuurs S.B., Zekveld-Vroon R.C., Collin R.W., De Baere E., Koenekoop R.K., van Schooneveld M.J., Strom T.M. (2009). Homozygosity mapping reveals pde6c mutations in patients with early-onset cone photoreceptor disorders. Am. J. Hum. Genet..

[30] Nishiguchi K.M., Sandberg M.A., Gorji N., Berson E.L., Dryja T.P. (2005). Cone cgmp-gated channel mutations and clinical findings in patients with achromatopsia, macular degeneration, and other hereditary cone diseases. Hum. Mutat..

